# Pro-ana versus Pro-recovery: A Content Analytic Comparison of Social Media Users’ Communication about Eating Disorders on Twitter and Tumblr

**DOI:** 10.3389/fpsyg.2017.01356

**Published:** 2017-08-11

**Authors:** Dawn B. Branley, Judith Covey

**Affiliations:** ^1^Department of Psychology, Durham University Durham, United Kingdom; ^2^Department of Psychology, Durham University Stockton-on-Tees, United Kingdom

**Keywords:** eating disorders, internet, pro-ana, social media, communication, mixed methods, individual differences, gender

## Abstract

**Objectives:** To compare how people communicate about eating disorders on two popular social media platforms – Twitter and Tumblr.

**Materials and Methods:** Thematic analysis was conducted to characterize the types of communications posted, and a content analysis was undertaken of between-platform differences.

**Results:** Three types of content (pro-ana, anti-ana, and pro-recovery) were posted on each platform. Overall, across both platforms, extreme pro-ana posts were in the minority compared to anti-ana and pro-recovery. Pro-ana posts (including ‘thinspiration’) were more common on Twitter than Tumblr, whereas anti-ana and pro-recovery posts were more common on Tumblr.

**Conclusion:** The findings have implications for future research and health care relating to the treatment and prevention of eating disorders. Developers of future interventions targeting negative pro-ana content should remain aware of the need to avoid any detrimental impact on positive online support.

## Introduction

Online communities which potentially encourage eating disorders (ED) are a cause of public concern (Tong et al., 2013) and they have received heavy media attention over the last decade (e.g., Rojas, 2014). Social media has made it easier for messages which encourage or promote ED to spread; this may have negative effects on vulnerable individuals ranging from healthy individuals who may be influenced to engage in ED behaviors, through to the ‘triggering’ of individuals who may already have an ED (Brotsky and Giles, 2007; Rouleau and von Ranson, 2011; Bond, 2012). Recent research suggests a link between viewing of online ED content and offline ED behavior (Branley and Covey, 2017), although the nature of this relationship is currently unknown. Of particular concern are ‘pro’ communities that arguably aim to encourage ED behavior. For example, there are many pro-ED (also known as pro-anorexia or pro-ana) communities that construe ED as a lifestyle choice rather than disorders to be treated (Wilson et al., 2006; Bond, 2012; Syed-Abdul et al., 2013; Tong et al., 2013). By encouraging each other to engage in associated ED behaviors, pro-communities normalize the behavior by making the user feel that it is acceptable, justifiable, and sometimes even desirable (Schroeder, 2010). Similarly, media portrayal of celebrities with perceived ED has been liked to an online practices around ED (Yom-Tov and Boyd, 2014). Another concern is the potential for pro-communities to glorify or romanticize ED, for example by portraying the behaviors as ‘tragically beautiful’ (Bine, 2013). Concerns are heightened further due to the interactivity of social media, (Borzekowski et al., 2010) and its heavy use by young users – the age group most affected by ED (Arcelus et al., 2011).

These concerns generally result in a call for interventions based upon censorship, i.e., removal of ED content from the platform (Tong et al., 2013) or warning messages or warning messages (online alerts displayed to users explaining that content associated with ED keywords or search terms can have the potential to be upsetting and/or triggering). However, these generic messages appear regardless of the type of ED content actually being returned by the search and users have to bypass this message even if searching for positive content (e.g., seeking information regarding recovery). This could render the warning meaningless. Censorship of ED content could also backfire; social media use has been linked to mental health wellness through contribution to perceived social support (Asbury and Hall, 2013) and not all online communities encourage ‘pro’ attitudes toward ED. Many feature information on recovery and some communities are supportive of users who decide to seek treatment (Brotsky and Giles, 2007; Csipke and Horne, 2007; Lipczynska, 2007). Therefore it is possible that social media provides a platform through which users can find help and guidance – this is particularly important as ED sufferers rarely seek professional help (Cachelin and Striegel-Moore, 2006). If there are positive elements to social media, then censoring content without any regard for the impact of the loss of positive social connections could have a negative effect upon users’ wellbeing.

It is important therefore that we truly understand the nature of online communities before introducing interventions. In particular, we need to know more about the types of ED-related information people using social media are being exposed to. However, our understanding is limited because previous researchers investigating online ED content have tended to focus upon pro-ED content and not ED-related content more generally (Norris et al., 2006; Brotsky and Giles, 2007; Lipczynska, 2007; Tong et al., 2013). The current study addresses this limitation by analyzing the full range of ED-related content shared by social media users. With no restrictions this analysis provides a balanced insight into the different ways in which ED is portrayed on social media. Focus is placed upon comparing the content on two popular social media platforms that have been linked to disordered eating behaviors in the press, Twitter, and Tumblr (e.g., “Becoming what you don’t eat” [Twitter], The Daily Iowan, June 26, 2014, “The hunger blogs: A secret world of teenage thinspiration” [Tumblr], Huffington Post, September 2, 2012). Whilst Twitter and Tumblr are both blogging platforms they differ in their functionality and the environment they create for users therefore allowing us to investigate whether the environment that is provided by the platform may impact upon the type of content shared by users. For example, users often perceive Tumblr as more private in comparison to Twitter therefore it is possible that this may influence the type or severity of the content shared (Marwick and Boyd, 2010).

## Materials and Methods

The research was reviewed and approved by the Durham University Institutional Review Board. Data was collected from Twitter and Tumblr from a 24-h period on June 12, 2014 providing a comparative analysis of ‘one day in the life’ of ED-related posts on the two platforms. The date was selected randomly from all dates within a 3 month period in 2014 (May to July). However, to ensure that the selected day was representative of a normal or typical day, dates within 2 days of a recognized holiday were not included in the selection particularly as religious holidays might impact upon eating behavior.

Due to public API’s imposing limitations on the data that they can provide (Morstatter et al., 2013; González-Bailón et al., 2014), the Firehose was used to access the Twitter and Tumblr data. The Firehose provides full, unlimited access to the complete database of publicly available data on the platforms. Access to the Firehose was obtained using Topsy (Twitter data) and DiscoverText (GNIP Firehose for Tumblr data).

The website www.hashtagify.me was used to identify the 10 most commonly used hashtags (#) relating to each of the following ED terms: anorexia, ED, proana, pro-ana, pro-ed, and proed. Although hashtagify.me is a Twitter specific search engine, it can still be useful when trying to identify search terms in general – at least in the initial planning stages. Using the list of terms gathered using this technique, the researcher DB manually searched for each of these terms on both the Twitter and Tumblr platforms and noted any other ED-specific terms which had not been captured. This produced a list of 55 potential search terms. In order to narrow this down to the most relevant search terms, each of the terms were entered into Topsy which provided an estimate of the daily number of tweets. Any terms showing a frequency of less than 100 tweets per fortnight were excluded from the list of terms used for data collection shown in **Table 1**.

**Table 1:** Search terms used for data collection of Twitter and Tumblr posts.

Included	Excluded
proana/#proana/pro-ana/pro ana	amzn/amazon
anorexia/#anorexia	#porn
anorexic/#anorexic	#xxx
#promia	#sex/#sexual
bulimia/#bulimia	#thinebook
bulimic	30 days to thin
#eatingdisorder/eating disorder	skinny dip/skinny dipping
#edproblems	#adult
ednos/#ednos	shop now/buy now
thinspiration/#thinspiration	
thinspo/#thinspo	


*Twitter posts collected using Topsy, Tumblr posts collected using DiscoverText (GNIP Firehose)*.

The searches conducted using the terms shown in **Table 1** produced a database of over 12,000 tweets and 73,000 tumblogs. An inductive, thematic approach to the analysis was adopted to identify patterns or themes within the posts (Braun and Clarke, 2006; Joffe, 2011). This approach has the ability to identify manifest and latent motivations that drive behavior (Joffe, 2011); therefore helping to achieve the goal of understanding ED communities from the perspective of the users (Vaismoradi et al., 2013). Posts were randomly selected from each database until data saturation was reached and no new themes were obtained. Posts that were clearly spam or not written in English were excluded from the analysis.

The themes identified from the thematic analysis were used to develop the coding scheme for content analysis. The coding scheme was tested for inter-rater reliability on a randomly selected subsample of 80 posts. The coding scheme was refined and repeatedly tested on new subsamples of posts until all codes had a Kappa value greater than 0.70. The final coding scheme is shown in **Table 2**. The content analysis was then conducted on a new random sample. The aim of this analysis was to compare the content between the platforms; power analysis confirmed that a sample of 350 posts from each platform was sufficient to detect a small effect size using Chi-square tests (power = 0.80, α = 0.05, φ = 0.12).

**Table 2:** Coding scheme for content analysis of Twitter and Tumblr between-platform differences.

Code		Twitter (*N* = 353)	Tumblr (*N* = 356)	χ^2^
Location where post originated	United States	78 (22.1%)	33 (9.3%)	ns
	United Kingdom	19 (5.4%)	12 (3.4%)	ns
	Canada	6 (1.7%)	5 (1.4%)	ns
	Germany	5 (1.4%)	5 (1.4%)	ns
	Other	19 (5.4%)	9 (2.5%)	ns
	Unknown	225 (64%)	292 (82%)	ns
Gender	Male	52 (14.7%)	20 (5.6%)	*p* < 0.001
	Female	249 (70.5%)	196 (55.1%)	ns
	Organization (charity or business)	15 (4.2%)	2 (0.6%)	*p* < 0.001
Identifiable	Full name provided	112 (31.7%)	15 (4.2%)	*p* < 0.001
	Identifiable from photo	116 (32.9%)	90 (25.3%)	ns
Original post written by user		213 (60.3%)	47 (13.2%)	*p* < 0.001
Nature of post	Pro-ana	91 (25.8%)	49 (13.8%)	*p* < 0.001
	Anti-ana	95 (26.9%)	175 (49.2%)	*p* < 0.001
	Pro-recovery	38 (10.8%)	50 (14%)	ns
Body images and nature of image	Thinspiration	32 (9.1%)	44 (12.4%)	*p* < 0.001
	Challenged norm around thinness as the ideal	1 (0.3%)	95 (26.7%)	*p* < 0.001
	Body image shared (unclear purpose)	4 (1.1%)	19 (4.8%)	ns
Emotion expressed	Negative emotion	28 (7.9%)	23 (6.5%)	ns
	Positive emotion	7 (2%)	3 (0.8%)	ns
	Mixed emotion	1 (0.3%)	9 (2.5%)	*p* < 0.01
Support offered and nature of support	Pro-ana support	3 (0.8%)	3 (0.8%)	ns
	Anti-ana or pro-recovery support	29 (8.2%)	35 (9.8%)	ns
	Support offered (unclear purpose)	2 (0.6%)	3 (0.8%)	ns
Raising awareness	Whether the post appeared to aim to raise awareness of issues around ED in others.	35 (9.9%)	53 (14.9%)	*p* < 0.05
Challenging norms	Whether the post appeared to be aiming to challenge social norms, e.g., pressure to be thin.	46 (13%)	134 (37.6%)	*p* < 0.001
Humor	Whether the post referred to ED in a humorous manner.	96 (27.2%)	64 (18%)	*p* < 0.01


## Results

### Thematic Analysis

One hundred and ninety posts were sampled to reach data saturation in the thematic analysis. In order to ensure user anonymity no user IDs, names or profile photos are included in the results. Also, any posts referred to in the results are paraphrased to prevent possible user identification.

Three distinct types of posts were identified from the thematic analysis: pro-ana, anti-ana, and pro-recovery. For the purpose of this research and in keeping with the context in which the term is commonly used, ‘pro-ana’ refers to all pro-ED content including that which encourages bulimic or other disordered eating behaviors and is not necessarily specifically about anorexia nervosa.

#### Pro-Ana: ‘Thinspiration’

Pro-ana posts included content that depicted a desire to engage in ED behaviors without any indication that recovery was desired and no apparent recognition of disordered eating as a negative behavior. Some users who made pro-ana posts appeared to judge their ‘success’ depending upon how little they had eaten or how long they had fasted. Emphasis was placed upon having self-control over hunger and doing so was regarded as an achievement. Signs of hunger were also indicators of success, i.e., “the sound of a stomach rumbling is equivalent to the sound of applause.” Some pro-ana posts offered or requested support to encourage ED behaviors, e.g., tips on prolonging fasting, and overcoming faintness from lack of food.

Eating disorders were portrayed as a lifestyle choice in some posts with users sharing ‘motivational’ material, e.g., images of tape measures and quotes such as “keep going, nothing tastes as good as skinny feels.” Motivational messages featured so-called ‘thinspiration’ or ‘thinspo’ images of extremely thin women displaying extremely protruding collarbones, hipbones and ribs, or thigh gaps. One reoccurring quote was seen on numerous Tumblr posts: “feet together, thighs apart.” The most severe ‘thinspiration’ images featured hashtags such as #bones, #ribs, and #sexy and were sometimes referred to as ‘bonespiration’ or ‘bonespo.’ Interestingly no male ‘thinspiration’ images were found during the analysis.

Some posts expressed negative emotions such as sadness, frustration, anger, and self-dissatisfaction. One user stated “When I look in the mirror I see nothing good, nothing attractive. I’m fat, I don’t have perfect arms/collarbones/legs/cheekbones, etc.” whilst another posted feelings of “wanting to die” because she had binged. Whilst still taking a largely pro-ana stance, some users depicted a love-hate relationship with their ED. One user stated that she did not understand how her ED could make her feel “so strong” yet it was actually making her weak.

Rather than simply posting pro-ana content occasionally, some users had blogs which were dedicated to pro-ana content. Some users used their blog to document how much they ate, drank, and exercised. Their biographies or ‘about me’ sections often including a list of ‘goal weights’ with many users choosing to record their progress. Some users shared progress pictures (‘body shots’) to document their weight loss, sustain motivation, and/or receive encouragement from others. Generally, the dedicated pro-ana blogs seemed to have a gloomier feel than other blogs, particularly on Tumblr where greater user customisation is possible. Many displayed only black and white content and featured angsty music. Many blogs also referred to the user experiencing depression, suicidal ideation, self-harm, and other mental health disorders.

#### Anti-ana: Challenging the Glorification of ED

Some posts expressed an explicit resistance against the pro-ana mentality, challenging the sharing of pro-ana material and expressing concerns regarding the potential harm it may elicit in vulnerable users. Anti-ana users were generally not against discussion about ED providing this was done in a positive manner, e.g., helping to alleviate feelings of isolation. However, they did not agree with any content that encouraged ED or portrayed it as a lifestyle choice. They expressed concern that pro-ana content could trigger the development or worsening of ED (including relapse from recovery) in vulnerable users. Some anti-ana users had ED themselves, e.g., one user blamed the sharing of ‘thinspiration’ and pro-ana diets with fueling her ED. Others expressed concern at how easy it is to ‘stumble upon’ potentially harmful ED content on social media without even explicit searching. Concerns were also raised regarding the online glorification of ED, stating that such behaviors are sometimes worryingly portrayed as ‘tragically beautiful’ or ‘trendy.’

In contrast to pro-ana content, blogs by users who shared anti-ana content were the most diverse. These users did not have blogs dedicated to sharing ED-related content and do not appear to be using social media solely to challenge pro-ana material.

#### Pro-Recovery: Sharing and Inspiring Recovery

Pro-recovery posts encouraged recovery in the user or others. This included posts that shared the user’s own recovery progress and struggles. For example, one user wrote about how she now has a healthy body with no thigh gap or protruding hipbones but she was feeling nervous about going to the swimming pool with her friends as she thought she would still feel that her body was not “pretty enough.” She mentioned that her friends have told her she looks better and that this makes her happy, but she appeared to be seeking reassurance from other social media users. Other posts aimed to inspire recovery in others, providing reassurance that recovery is possible. Some posts shared advice on how to combat disordered eating habits and stick to a healthier lifestyle. For example, one user advised another that it is important to stick to regular meal times; saying that “no good” comes from counting how many calories are in everything. Other users suggested healthier ways to keep in shape such as healthy levels of exercise rather than extreme dieting. Some users simply offered support in the form of empathy, compassion, and understanding.

The daily conflict between their desire to engage in ED behavior and the desire to recover was highlighted. One user described gaining weight due to her recovery, and although she ultimately thought of this as something to be proud of, another part of her was sad because that part of her still really wanted to lose weight. Similar mixed emotions were portrayed by a user who described how her dietician had informed her that she was now in the healthy weight range and that this “freaked her out” because she knew that she would begin to obsess about her weight.

Similar to those found for pro-ana, some blogs were dedicated to sharing pro-recovery content. With the exception of a minority of blogs belonging to ED charities and organizations, pro-recovery blogs appeared to be run by users currently in recovery from their own ED. In stark contrast to the pro-ana blogs, pro-recovery blogs tended to be colorful with positive imagery and slogans such as ‘life can be wonderful’ and ‘recovery is possible.’ Pro-recovery bloggers appeared to be aiming to inspire a more optimistic mood for themselves and/or others.

#### Raising Awareness, Challenging Social Norms and the Trivialisation of ED

Amongst the users sharing anti-ana and pro-recovery posts, two shared motivations became apparent: raising awareness about ED among the wider population (e.g., by sharing content from online newspaper articles, TV documentaries and charity organizations), and challenging social norms by expressing dissatisfaction with the social and cultural emphasis that is placed upon being thin.

The search also identified blogs in which ED terms were used in humor. Often ED terms were used in a flippant manner for example in one post ‘anorexia’ was used as a substitute for the word ‘empty,’ e.g., “my kitchen cupboards are so empty they’re anorexic.” In other instances, ED terms were used as ‘playful’ insults between friends, e.g., one male user sent a tweet to his male friend calling him a “bulimic bitch,” whilst another user tweeted “[@username] is so thin I named my ED after her.” Although both recipients retweeted and liked/favorited the posts indicating that they had been received as humorous and not hurtful, others may view these posts as trivializing a serious disorder. A popular reblogged post asked others not to make jokes about ‘thinspiration’ and online ED communities, stating “do not make fun of something you do not understand.”

### Content Analysis

The coding scheme that was developed from the thematic analysis and applied to a random sample of 709 posts from Twitter (*N* = 353) and Tumblr (*N* = 356) is shown in **Table 2**. The coding scheme also identified (if possible) the location from which the post originated, the gender of the user, whether they could be identified (either from their name or photograph), and whether the post was written by the user themselves.

Chi-square tests were conducted to test for significant differences between the platforms. As shown in **Table 2**, this analysis showed that the majority of users posting ED-related content on both platforms were female, although posts from males were more common on Twitter [*X*^2^(1) = 10.57, *p* = 0.001, φ = 0.13]. Posts from organizations were also more common on Twitter [*X*^2^(1, *N* = 619) = 8.12, *p* = 0.004, φ = 0.12], and Twitter users could often be identified from their full name [*X*^2^(1) = 76.51, *p* < 0.001, φ = 0.36]. These findings are in keeping with previous research, which has shown that Twitter is used as a broadcast medium where a lack of anonymity is the ‘norm’ whereas Tumblr tends to be used more anonymously by users and screen names/aliases are more common (Marwick and Boyd, 2010).

In terms of the types of ED-related posts on the two platforms it was notable that pro-ana posts were more common on Twitter [*X^2^*(1) = 31.54, *p* < 0.001, φ = 0.25], whereas anti-ana posts were more common on Tumblr [*X^2^*(1) = 22.86, *p* < 0.001, φ = 0.21]. Nearly half of the posts (49.2%) on Tumblr were anti-ana and in combination with pro-recovery posts made up the majority of posts (63.2%).

Body images were more likely to be included in the posts shared on Tumblr [*X*^2^(1) = 102.55, *p* < 0.001, φ = 0.38], which is not unexpected given its more visual nature. Although Twitter users were sharing fewer images, of those users who *did* share body images, these were more likely to be shared for ‘thinspiration’ (pro-ana) reasons [*X*^2^(1) = 42.55, *p* < 0.001, φ = 0.47]. In comparison, there was a greater proportion of Tumblr users sharing anti-ana images which challenged social norms about thinness [*X*^2^(1) = 40.51, *p* < 0.001, φ = 0.46]. Whether images were used or not Tumblr users were generally more likely to post content challenging norms compared to Twitter users [*X*^2^(1) = 58.84, *p* < 0.001, φ = 0.29]. Twitter users were, however, significantly more likely to refer to ED in humor [*X*^2^(2) = 16.41, *p* = < 0.001, φ = 0.15]. It is possible that gender differences in the platform user group may contribute to these findings. As shown in **Table 2**, the Twitter posts were more likely to come from males than the Tumblr posts and testing for gender differences in the use of humor revealed a borderline significant result for males showing greater use of ED-related humor than females [*X*^2^(1) = 3.85, *p* = 0.050].

## Discussion

The current study is the first of its kind to look in detail at publicly accessible ED-related content on two social media platforms – Twitter and Tumblr. Three distinct types of posts were identified from the thematic analysis: pro-ana, anti-ana, and pro-recovery. Encouragingly, pro-ana was in the minority compared to the latter two groups. That said, the potential impact of pro-ana posts should not be dismissed. The thematic analysis illustrated the extreme content of some of the pro-ana posts on both platforms including ‘thinspiration’ images that may have the potential to trigger disordered eating behaviors in vulnerable users. Many users recognized this risk and included ‘trigger warnings’ alongside their posts (or as hashtags, e.g., #TW). Trigger warnings are a statement or keyword that warns the viewer that content on the blog may encourage disordered eating in vulnerable users. However, it has been suggested that the use of trigger warnings may help users to purposefully search for pro-ana content (Borzekowski et al., 2010) and the use of hashtags is likely to facilitate this.

Some of the users who posted pro-ana material referenced their own depression and suicidal ideation which supports research which has shown negative affect to be comorbid with ED (Braun et al., 1994). It is still an open question, however, whether social media use impacts upon users’ mental and emotional wellbeing. A recent meta-analysis suggests that exposure to online content depicting ED is linked to negative affect and negative body image (Rodgers et al., 2016) – although identifying causality is difficult in correlational studies. Vulnerable users viewing or sharing pro-ana posts may be more influenced by the content that they see online due to their current state of mind. Therefore, some populations may be more at risk than others. Further research is required to investigate this further.

There are some similarities between our findings and those from previous research which has investigated ED content on more traditional Web 1.0 websites and forums. For example, we also found ‘thinspiration’ photos, content challenging social norms around unrealistic ideals and a mix of pro-ana and pro-recovery content (Borzekowski et al., 2010). However, contrary to Web 1.0 pro-ana websites (Bond, 2012) we found very little evidence of competition amongst the users, i.e., to be the most ‘successful ana.’ There was also a lack of content showing the ‘religious-type quality’ found by Bond (2012), i.e., portraying ED as akin to a religion. This suggests that social media may not feature content as extreme as dedicated pro-ana websites. This could be due to social media feeling more ‘public’ compared to the privacy of dedicated websites (Branley, 2015).

Most of the ED-related content was posted by females on both platforms. They also shared more pro-ana content than males. This may be a consequence of ED prevalence being higher amongst females (Fairburn and Harrison, 2003; Hudson et al., 2007; Preti et al., 2009), females being the most predominant users of social media, (Branley, 2015), and/or female users being more likely to use social media to communicate about ED. Branley (2015) found that females are more likely to use social media for social reasons and therefore they may be most likely to turn to this platform for support. It is also possible that males are using social media but not revealing gender identifying information on their account (perhaps due to worries over stigma) and/or that males tend to browse ED content rather than actively participate in sharing content themselves. Further research including interviewing participants with ED may be able to identify whether social media play a different role for males and females.

The content analysis showed between-platform differences in the prevalence of each type of post. It was perhaps surprising to find more pro-ana material on Twitter considering that this platform is generally regarded as more public and therefore less suitable for expressing potentially controversial opinions (Marwick and Boyd, 2010). However, it is possible that one platform was sharing more extreme pro-ana material than the other. For example, although there were more pro-ana posts on Twitter, our analysis suggested that Tumblr posts were more often presented within blogs that had been customized with black and white visuals or ‘gloomy’ music.

The content analysis also showed that Tumblr had more anti-ana content than Twitter. This shows that pro-ana content is met with negativity and does not go unchallenged by Tumblr users in particular. Many users expressed feeling pressure to conform to the cultural norm of thinness as the ideal. Previous research suggests that disordered eating can be caused or exacerbated by cultural pressures that glorify the “perfect body” (Lewinsohn et al., 2002). It is possible that these cultural ideals are being communicated by social media and may affect vulnerable users. Further research is necessary to investigate whether users feel social media is contributing to these social pressures or whether they regard it as a helpful platform through which societal norms can be challenged. Such a function was particularly notable on Tumblr with over a third of posts challenging the pressure to be thin.

There was also evidence of pro-recovery content on both platforms with 10.8% pro-recovery posts on Twitter and 14% on Tumblr. Such posts included messages providing support and empathy to others experiencing ED on both platforms. Although these findings illustrate the positive functions of ED-related content on social media further investigation is required to identify whether the social support offered has a positive or negative effect upon users (Juarascio et al., 2010).

One of the few studies to look at ED content on social media is that by Teufel et al. (2013), however, again they investigated a more private element of social media by analyzing ED content in private Facebook groups, i.e., groups on Facebook where users have to ‘request’ to join. Therefore, only those Facebook users who are members of that specific group will see the group content. In contrast, we were interested in the type of ED content that was available more widely, i.e., publicly on social media platforms. Differences aside, the study by Teufel et al. (2013) also found that social support appears to be a key element in the communication around ED online. However, they reported a lack of professional, pro-recovery content on Facebook – something that was echoed in our own results Whilst pro-recovery and anti-ana content was found on both platforms, this tended to be from other users rather than professional sources, e.g., charities or health organizations. Social media provides a potential haven of social support for users experiencing (or at risk of developing) disordered eating behavior. This suggests that there may be potential for these platforms to be used for positive reasons such as offering appropriate support and advice, and/or identifying users who could benefit from guidance toward help sources. Presently the only widely available support on social media appears to be from other users – increasing the risk of support being received from detrimental sources. Future research should seek to investigate the potential to develop positive online interventions and/or the strengthen the presence of positive support sources on public social media platforms. Alternatively, improved methods of content moderation (rather than censorship) may be useful.

Social media is an integrated part of modern day life, especially amongst the age cohort most susceptible to the development of ED. Therefore therapists, caregivers and other health professionals should be provided with detailed information regarding the type of content users can find online and guidelines on how to approach and address these issues with patients, friends, and family members. Greater awareness may be the first step toward limiting the potential negative influences of social media use. In addition, discussing patients/family members’ social media use may provide a gateway to discuss why individuals are motivated to talk about this behavior online. This could help to identify informational and/or interpersonal needs, which are not currently being met in the offline environment. This is an area of research in its infancy and there is much potential for future researchers.

Content analysis of social media data can be problematic due to the expanse of data connected to every tweet and blog. Many tweets and blogs form part of a much larger conversation, just as users act in many different networks and capacities. It is not possible to capture or analyze all social media data therefore researchers should endeavor to analyze only the content captured by the data collection and resist extrapolating or speculating about what this content may relate to on a larger scale. For this reason, the inductive approach used in this research is recommended; this approach allows themes to emerge from the data rather than approaching data with pre-determined hypotheses or themes which may increase chances of misinterpretation. It is also important to remain aware that a certain degree of trolling and roleplay does still exist even within social media networks that are regarded as largely grounded in reality (i.e., those that are intended to represent the users real identify rather than a fantasy character, such as those in virtual game worlds, e.g., World of Warcraft). Researchers should stay aware that not all user posts will be truthful. Future research may wish to investigate the use of computational methods to investigate the trustworthiness of social media data [similar to link analysis that is used to determine the trustworthiness of webpages for page rankings (Ravikumar et al., 2012)]. Issues around trustworthiness does not only apply to researchers wishing to analyze social media data, perceived trustworthiness can also affect how users’ respond to tweets – something that health professionals should also consider if/when trying to improve their presence on social media platforms.

In summary, this study is the first to investigate the types of ED-related posts available publicly on social media and to incorporate between-platform comparisons between two of the largest platforms. The results inspire hope that there are positive elements to online communication about ED such as inspiring recovery, raising awareness, and challenging societal norms. However, it is vital to ensure that pro-ana content is not trivialized or dismissed. Researchers, health professionals, policy makers, and platform developers should aim to identify and develop approaches which limit negative content whilst minimizing disruption of potentially positive social networks/content. Future research should incorporate a measure of content severity and not restrict analysis to prevalence. User interviews would also be beneficial to establish how ED content is experienced and perceived and the impact that users feel this has in relation to their ED-related thoughts and behavior (including recovery).

## Ethics Statement

This study was carried out in accordance with the British Psychological Society recommendations for online research. The protocol was approved by the Durham University ethics committee.

## Author Contributions

DB was involved in all design of the project and conducted all acquisition, analysis, and initial interpretation of the data. JC contributed to project design, analyses, and interpretation. DB drafted the initial manuscript. Both authors contributed to reviewing and amending the final version.

## Conflict of Interest Statement

The authors declare that the research was conducted in the absence of any commercial or financial relationships that could be construed as a potential conflict of interest.
